# Web-Based Computational Chemistry Education with CHARMMing II: Coarse-Grained Protein Folding

**DOI:** 10.1371/journal.pcbi.1003738

**Published:** 2014-07-24

**Authors:** Frank C. Pickard, Benjamin T. Miller, Vinushka Schalk, Michael G. Lerner, H. Lee Woodcock, Bernard R. Brooks

**Affiliations:** 1 Laboratory of Computational Biology, National Heart, Lung, and Blood Institute, National Institutes of Health, Bethesda, Maryland, United States of America; 2 Department of Natural Sciences, New College of Florida, Sarasota, Florida, United States of America; 3 Department of Physics and Astronomy, Earlham College, Richmond, Indiana, United States of America; 4 Department of Chemistry, University of South Florida, Tampa, Florida, United States of America; University of Wisconsin-Madison, United States of America

## Abstract

A lesson utilizing a coarse-grained (CG) G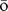-like model has been implemented into the CHARMM INterface and Graphics (CHARMMing) web portal (www.charmming.org) to the Chemistry at HARvard Macromolecular Mechanics (CHARMM) molecular simulation package. While widely used to model various biophysical processes, such as protein folding and aggregation, CG models can also serve as an educational tool because they can provide qualitative descriptions of complex biophysical phenomena for a relatively cheap computational cost. As a proof of concept, this lesson demonstrates the construction of a CG model of a small globular protein, its simulation via Langevin dynamics, and the analysis of the resulting data. This lesson makes connections between modern molecular simulation techniques and topics commonly presented in an advanced undergraduate lecture on physical chemistry. It culminates in a straightforward analysis of a short dynamics trajectory of a small fast folding globular protein; we briefly describe the thermodynamic properties that can be calculated from this analysis. The assumptions inherent in the model and the data analysis are laid out in a clear, concise manner, and the techniques used are consistent with those employed by specialists in the field of CG modeling. One of the major tasks in building the G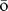-like model is determining the relative strength of the nonbonded interactions between coarse-grained sites. New functionality has been added to CHARMMing to facilitate this process. The implementation of these features into CHARMMing helps automate many of the tedious aspects of constructing a CG G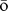 model. The CG model builder and its accompanying lesson should be a valuable tool to chemistry students, teachers, and modelers in the field.

## Introduction

To function properly, most proteins must fold [1]. Determining the structure and understanding the mechanisms responsible for folding are active areas of biophysical research, as gleaning this information may provide critical insights towards fighting diseases that have been linked to protein structure, such as Alzheimer's [2], [3]. Experimental determinations of protein structure are typically performed using X-ray diffraction of crystallized proteins or using NMR spectroscopy. Both techniques provide important information about a protein's native folded structure, yet both methods are not without their drawbacks. The process of crystallizing a protein is labor intensive, and structural information from a crystallized sample comes from a nonbiological environment. NMR studies on an aqueous sample yield time-averaged results and are unable to resolve many important dynamic details. Considering the limitations of these techniques, computer simulations are an important tool to supplement and interpret information provided through direct experiment. One way theoretical studies may lead to a better understanding of experimental results is by providing simple models with verifiable results.

**Figure 1:**
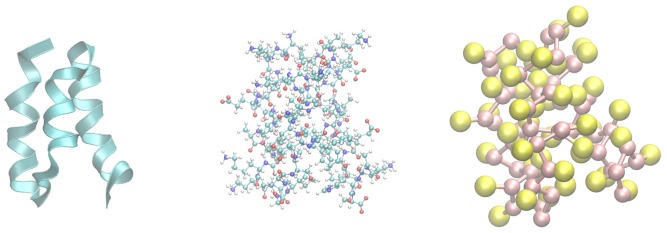
The native structure of the GA module of an albumin binding domain. Three representations of the structure are shown. The left panel shows the backbone of the native fold as a ribbon, which highlights the helical nature of the secondary structure. The center panel shows the AA structure, which is used to build the KT G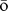 model (right panel).

Computer models can be constructed at a variety of scales or resolutions (Figure 1). Many simulation techniques represent each atom as a single interaction center. The interactions between beads can then be evaluated using a force field. However, it is possible to construct a coarse-grained (CG) model, in which multiple atoms are represented by a single center (as in the right-hand panel of Figure 1). These types of models can be useful theoretical tools because they can be specifically designed for a system or process of interest. By providing a simplified view of a complex molecular process, important physical details are retained in the model, while superfluous details are ignored. The resulting CG model distills the essence of the biophysical process into its most important physical details, allowing the computational scientist a fundamental understanding of the process.

The success of CG models in representing a variety of biological phenomena is widely acknowledged. Starting from the earliest protein folding simulations [4], to more recent studies on membranes [5] and transmembrane proteins [6], CG models have been applied to diverse phenomena such as protein aggregation [7], vesicle fusion [8], protein structure refinement [9], and the thermodynamics of RNA folding [10], among many other topics. For a thorough discussion of the many diverse applications of CG models, please see the recent review literature [11]–[13].

Besides their simplification of complex biological phenomena, another attractive feature of CG models is their inexpensiveness relative to all-atom (AA) models. The computational cost of performing a classical molecular mechanics simulation is typically dominated by the calculation of nonbonded forces (electrostatic and van der Waals interactions), as the number of interaction pairs is proportional to the square of the number of particles present in the model system. For example, one of the most widely-used AA water models, TIP3P [14], represents a water molecule as three interaction sites. By comparison, the MARTINI model [5] represents four water molecules using a single CG site, an effective reduction of 12 sites into one. If a ten-to-one mapping is assumed for an entire CG model, the number of required nonbonded interactions to be computed is reduced by roughly two orders of magnitude.

One commonly used CG model is the G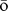-like model [15]. In this type of model, an AA crystal structure is used not only to build the coordinates and topology of the CG model (as is the case with most CG models) but also to build the parameters for the non-bonded interactions. The assumption underlying the G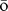 model is that the native state below the melting temperature (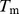) is the global free energy minimum. We know that the protein folds into a given crystal structure; therefore, we assign parameters to always reproduce the experimentally known fold. Because we know the native state is stable, we know that native contacts are more stable than non-native contacts. Therefore we assign an attractive interaction potential to native contacts, and a short range repulsive interaction potential to non-native contacts. While the G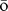 model is not based on first principles, nor is it transferable (like the AA protein CHARMM force field) [16], these simulations reach equilibrium (for small globular proteins), allowing for direct comparison between simulation and experiment [17]–[20].

G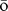-like models have been used to describe the kinetic features of protein folding [21]. Among the systems studied are the Trp-cage fast folder [22], the Villin headpiece [23], ribosomal protein S6 [24], and c-src SH3 and CI2 [25]. Various physical insights have been gained from these studies; however, the performance of a particular model depends upon how accurately it can reproduce AA kinetics. Some investigation of optimal parameters has been made [26]. An accurate G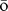-like model can provide these insights at a much smaller computational cost than its AA counterpart at the expense of transferability and fine-grained insights into structural behavior.

A consequence of coarse-graining the molecular model is the smoothing of the interaction potential. This CG potential has two important implications: larger integration time step and accelerated dynamics. In the reductive mapping process, many lighter particles are lumped together to form massive interaction centers; this effectively integrates out the fastest moving degrees of freedom, allowing the usage of a longer integration time without incurring integration errors (phantom heating). For example, in AA simulations the hydrogen stretching motions occur at 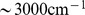, dictating an integration time step of 1 or 2 fs. By removing this high-frequency motion, one may safely use a longer time step. This allows a total dynamics simulation time one order of magnitude longer than that of an AA model over the same number of energy and force evaluations. The smoother interaction potential also indirectly accelerates MD by removing degrees of freedom, causing energetic barriers between conformations to disappear. This effect can accelerate the rate of biophysical processes by another factor of two [12]. Taken together, these effects can facilitate the simulation of many biological processes, such as protein folding, that would be impossible to rapidly simulate using AA dynamics. CG models are therefore very attractive in an educational environment, as they retain the qualitative features of their AA counterparts and can be performed cheaply and rapidly, leading to a far more interactive experience with the students.

In this lesson, we will use a G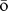-like model in which each amino acid is mapped into two CG sites, one located at the alpha carbon (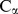) and one located at the side-chain center of mass (SC). Many G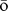-like models only provide for one interaction site per residue. One such popular model has been developed by Karanicolas and Brooks [27], [28]. A web server that generates CHARMM input files for this model is available. Users are not able to perform test runs of their models through the server itself, and there are no interactive lessons, making it less comprehensive than the current CHARMMing implementation. However, some tutorial materials are available from the Multiscale Modeling Tools for Structural Biology (MMTSB) website [29].

In the remainder of this work we describe some of the details underlying both the bonded and non-bonded forces in the G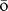 model potential. We also discuss the details of the dynamics simulations performed by CHARMMing [30] (www.charmming.org) using the CHARMM simulation package [31]. A procedure for extracting thermochemical values from the raw dynamic trajectories is also given. We then outline step-by-step directions for setting up, performing, and analyzing the dynamics simulation using CHARMMing. This manuscript will give a basic description of the procedure suitable for undergraduate students. New sections have been added to the CHARMM tutorial available at www.charmmtutorial.org with more in-depth descriptions of the methodology, and these are referenced within this work. Finally, we discuss the utility of this lesson both as an educational tool and as a research aid.

## Methods

Two CG protein models have been implemented into CHARMMing [30]. The one described in this manuscript is a two-site G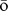 model based on the work of Klimov and Thirumalai and described further in this section [18]. The second model characterizes the chemical properties of residues and defines interactions accordingly. For the G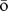 model, the bonded and nonbonded parameters are explicitly constructed to bias the protein towards the experimental crystal structure. However, the inclusion of a SC particle serves to hinder the progress down the folding funnel and frustrate the folding process. This creates a better balance between the folded and unfolded states than is typical in one-site G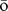 models such as the model of Karanicolas and Brooks referenced above, allowing for a more accurate description of folding thermodynamics. Further modifications to the standard G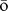 model allow the CG model to incorporate hydrogen bonding and secondary structure into the parameterization process.

The bonded and nonbonded force field terms of the two-site G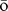 model use the same functional form as those used in the AA CHARMM force field [16]. However, the model must be re-parameterized to account for the fact that each amino acid is only represented by two CG beads. This parameterization exploits the nontransferability of the model; parameters are designed to reproduce the secondary and tertiary structure of the AA system. The default parameters for the model are described in detail on charmmtutorial.org; interested readers are encouraged to consult this page (http://charmmtutorial.org/index.php/Coarse_Grained_Go_Models) for more information and default values of these parameters. Those readers wishing to learn about the functional form of the CHARMM force field may visit http://charmmtutorial.org/index.php/The_Energy_Function.

The bonded parameters for adjacent backbone atoms are modified based on the secondary structure (as determined by the STRIDE program [32]) of the AA model. However, the bond strength is somewhat weaker than would be found between backbone atoms of an AA model since these “bonds” actually represent more flexible linkages between residues. In addition, because the entirety of the backbone is represented by a single bead per amino acid, there is little to prevent 180 degree rotations of the side-chain that, in an AA representation, would correspond to a chiral flip. This is a rare occurrence in nature because of the energy barrier involved, and therefore, the model adds a strong improper dihedral term to mimic this barrier.

All nonbonded interactions between sites on non-adjacent amino acids are described by a 12–6 Lennard-Jones (LJ) potential. There are no direct electrostatic components to the energy, however electrostatic effects are incorporated indirectly as will be discussed below. The LJ parameters used for SC particles depend on whether they form a native contact within the crystal structure, defined as occurring when their positions are within 4.5 Å of one another. The objective of any G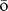-like model is to preserve such contacts while discouraging others. So in this case the LJ potential is attractive, with the minimum energy occurring when the SC beads are at the same distance as the SC centers of mass in the original crystal structure. The strength of the attraction is determined by an experimentally derived contact potential, the most widely used of which was developed by Miyazawa and Jernigan [33] (however, CHARMMing supports other contact potentials as well). This contact potential is how electrostatics are taken indirectly into account. All other SC–SC interactions are modeled with a non-attractive LJ potential, following the fundamental assumption of G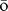 -like models that the native structure is most stable. All 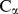
–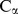
and 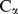
–SC interactions are also modeled non-attractively to account for volume exclusion, unless they take part in hydrogen bonding, in which case, the potential is slightly attractive. This method of simulating hydrogen bonds has been shown to produce qualitatively correct results for CG water [5].

One of the parameters of the Klimov-Thirumalai G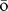-like model is the relative strength of the nonbonded versus the bonded interactions. The motivation behind this parameter, as described in the supporting information of [20], is that the strength of the native contacts must be scaled in order to provide a physiologically realistic melting temperature; however, the scale factor (nScale) is not known a priori. In the CHARMMing implementation, the user may specify nScale or ask the interface to estimate it. Our method of estimating nScale is to run temperature replica exchange [34], which gives a Boltzmann ensemble of the structure at a range of different temperatures. From these ensembles, a fraction of native contacts can be calculated, which can be used to plot a melting curve (see below), allowing the melting temperature (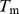) to be estimated. If 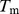 is close to a physiological value for the system of interest, then nScale is considered correct. Otherwise, it is strengthened or weakened depending on whether 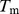 is too low or too high. A full discussion of temperature replica exchange is beyond the scope of this manuscript, but interested readers may consult http://charmmtutorial.org/index.php/Temperature_replica_exchange. If a user asks CHARMMing to determine nScale for them, the software takes an initial guess (the default being 1, no scaling), runs a brief temperature replica exchange simulation, and adjusts nScale upwards or downwards based on the 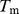 calculated from that simulation. The process is repeated until an nScale is found that yields a physiological 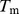.

Electrostatic interactions are not explicitly present in this model (they are accounted for implicitly through the LJ potential); commonly used implicit solvent schemes (e.g., Generalized Born) are fundamentally incompatible with this implementation of the G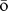 model. Solvation effects are incorporated into this model via Langevin Dynamics (LD) [35]. Both friction and knocking effects are approximately accounted for in this manner. CHARMMing allows the user to specify a collision frequency (damping coefficient), but 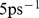 is employed in our lesson so as not to inhibit conformational transitions. The parameter files produced by CHARMMing set nonbonded cutoffs to 23 Å, with the standard CHARMM switching function operating beyond 18 Å [36].

## Data Analysis

**Figure 2:**
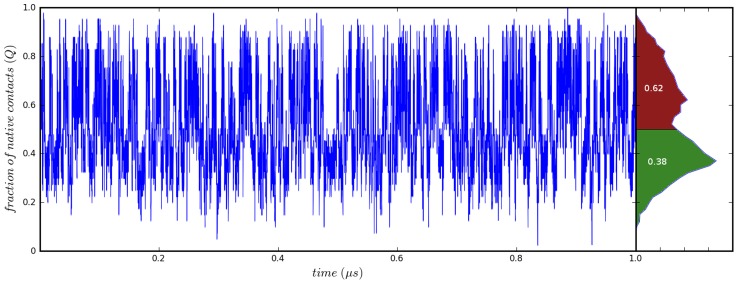
An example trajectory. The fraction of native contacts are plotted as a function of time. By inspecting the histogram at the right, we observe that this trajectory was simulated below 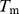, as the protein is folded for 62% of the simulation.

**Figure 3:**
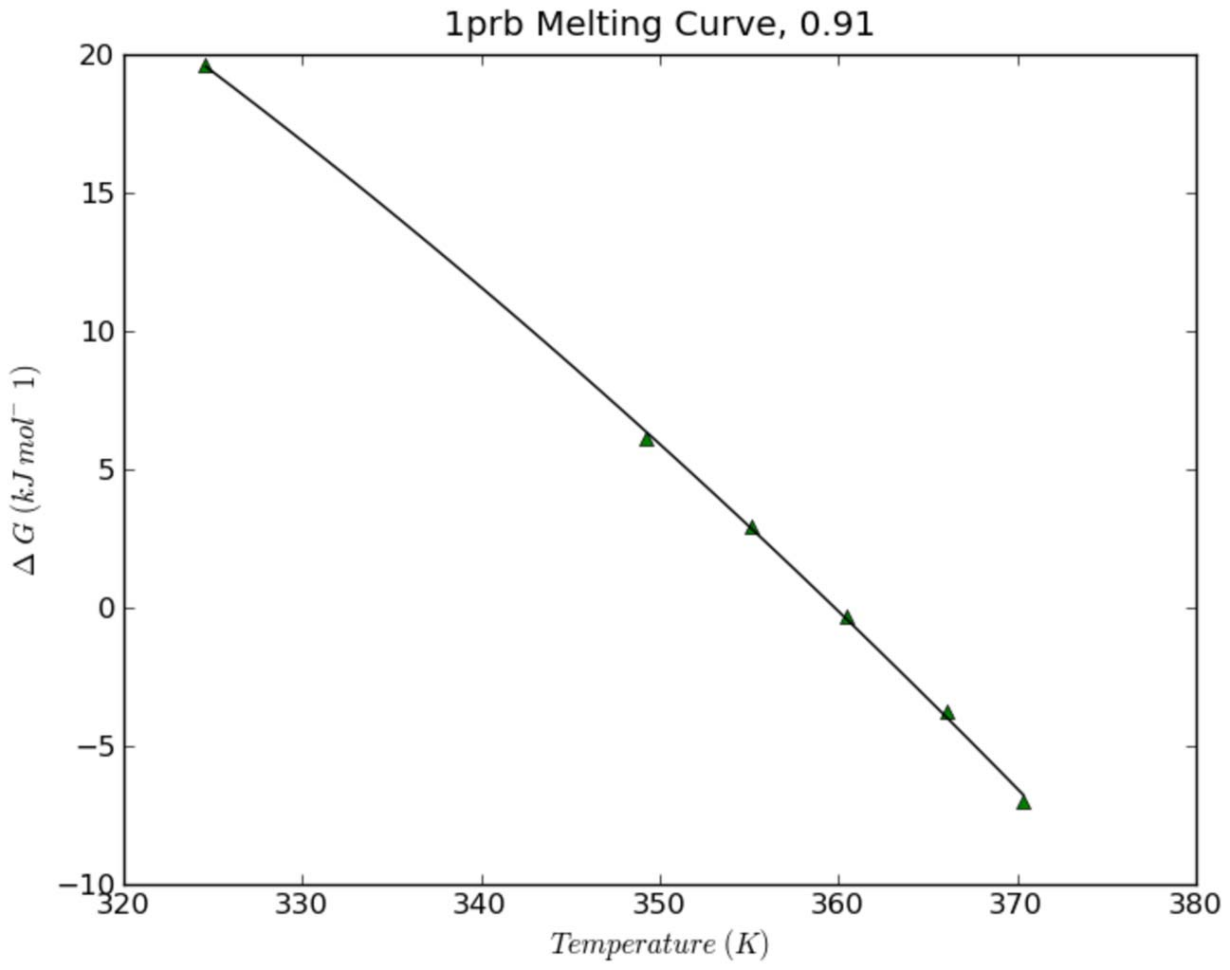
An example melting curve. Each point on the plot represents 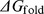 calculated from a full trajectory. 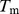 occurs when 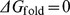, and the curvature of the plot is related to 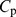.

When analyzing trajectory data, an important consideration is the choice of reaction coordinate employed in data analysis. As illustrated in Table 1, the choice of reaction coordinate can have an uncomfortably large effect upon the computed properties of the simulation. The choice of a reaction coordinate is very complex and depends heavily on what scientific questions are being asked about the system under study. Users are encouraged to consult the literature broadly when choosing a reaction coordinate for a new system. In this lesson, we will consider the fraction of native contacts (*Q*), a reaction coordinate which has been shown to be robust [37]. This reaction coordinate is often used when mapping the thermodynamic landscape of various folding pathways of a protein. When 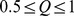, a protein is considered folded; when 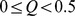, a protein is considered unfolded. The 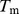 of a protein occurs when it is equally likely to be folded as unfolded. Figure 2 shows an example trajectory of a protein below its melting point. From the relative frequencies of folded versus unfolded structures, we can calculate 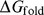. Furthermore, by considering how 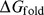 changes with respect to temperature, we can plot a melting curve and apply the Gibbs-Helmholtz equation to determine the protein's heat capacity (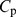) and enthalpy of fusion (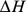) (see equation below). Figure 3 gives an illustrative example of a computed melting curve.

**Table 1:** 1PRB thermochemistry.

coord.	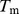	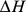	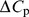
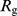	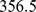	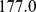	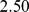
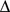	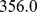	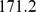	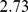
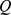	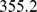	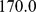	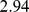
Expt. [44]	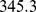	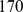	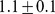
Expt. [45]	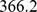	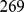	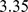

A comparison of three reaction coordinates, radius of gyration (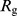), backbone root mean squared deviation (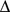), and fraction of native contacts (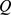), with experimental results [44], [45]. All results computed using 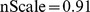.



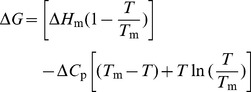



## The Coarse-Grained Lesson

**Figure 4:**
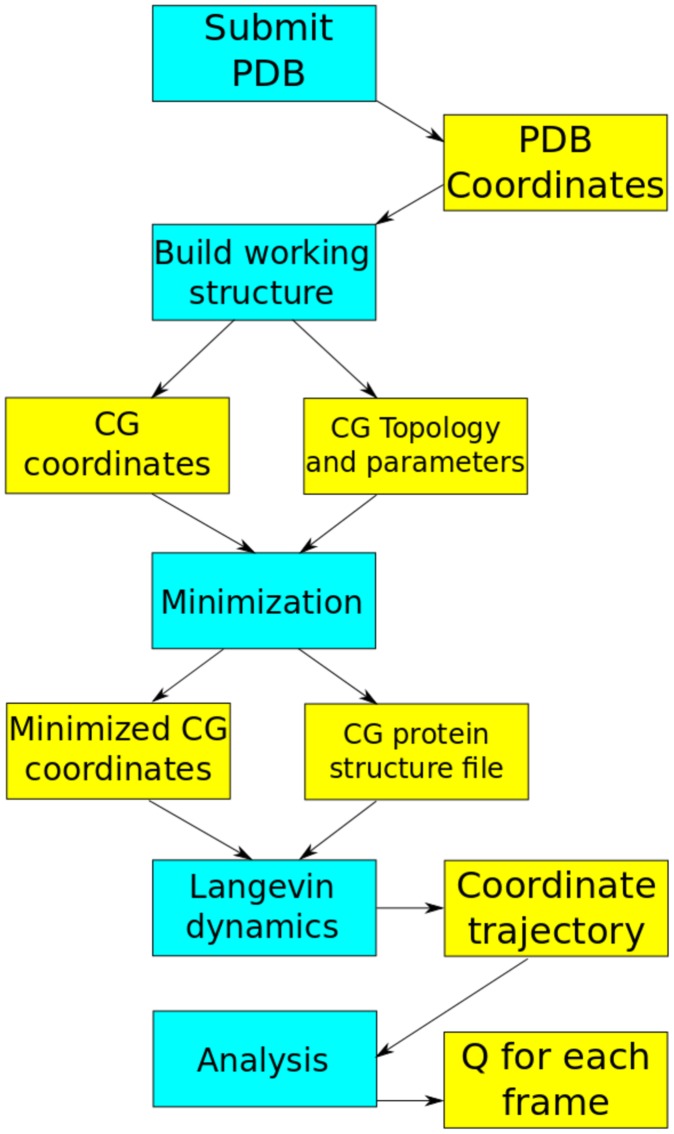
The workflow of the KT G 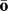 model lesson. Blue boxes represent the steps described in the text. Yellow boxes show the inputs and outputs of each step. Note that the CHARMM protein structure file is only built when a calculation is performed (minimization, in this case). *Q* is the fraction of native contacts as defined in the text.

In this lesson, we consider the protein G related albumin-binding domain (PDB code: 1PRB). This is a small globular protein that has been extensively studied, both experimentally and theoretically [37]–[39]. Experiment has shown that this protein folds near the semi-empirical speed limit of 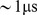 [40]. In solution, 1PRB forms a three-helix bundle (Figure 1); it readily interconverts between its folded and unfolded states, allowing rapid convergence of its thermodynamic properties. These features, taken together, make it an ideal candidate to demonstrate the efficacy of CG methodologies in an educational environment. The sequence of steps that are carried out by the lesson, along with their inputs and outputs, are shown in Figure 4.

### Steps 1 and 2: Upload a Protein to CHARMMing and Build the CG Structure

First CHARMMing must be directed to obtain an AA crystal structure for our protein of interest. The user must navigate to the “Submit Structure” page from the main menu. He or she selects “Retrieve a PDB using a PDB ID”, and enters the PDB code “1PRB” into the text box. For “What Lesson is this structure associated with?”, “Lesson 5” should be selected from the drop-down menu. The user then submits the structure. CHARMMing will now redirect to the page entitled “Build/Select Working Structure.”

On the “Build/Select Working Structure” page, an arbitrary name may be provided at the user's discretion to label the working structure. Next, the user must select what type of structure CHARMMing should build, select the radio button next to “A Klimov-Thirumalai style G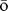 model” and indicate that the “a-pro” segment should be used to build the model by selecting the appropriate box. These options tell CHARMMing to construct a G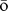 model with the appropriate CG topology, representing 1PRB. Also on this page, the user must tell CHARMMing how to generate parameters for the model system. We will use the Miyazawa-Jernigan statistical potential as the basis for generating the strengths of the native contact interactions. Under the “Contact types” heading, the “MJ” radio button should be selected. Next, the nScale parameter must be assigned; for 1PRB the optimal value is 0.91. It is important to note that the user would normally need to tune this value manually, as described above. The user can leave all other parameters at their default values and click “Submit.”

### Step 3: Light Minimization

Although the native crystal structure is determined to be at the free-energy minimum, we still ask the user to do a short energy minimization. This serves two purposes: firstly, it removes any minor issues with the geometry of the constructed structure, such as suboptimal non-native contacts or imperfect secondary structure, and secondly, it allows the user to verify that the model has been successfully built. In order to accomplish this, the user must go to the “Minimization” page and request ten steps of steepest descent minimization and 100 steps of adopted basis Newton-Raphson minimization. The process of minimization is described more extensively in the first article of this series and on charmmtutorial.org. Because a coarse-grained model has no high frequency motions, SHAKE, an algorithm used to constrain rapid bond and angle vibration [41], may be turned off.

### Step 4: Run the Langevin Dynamics Simulation

Once minimization is done, the user must go to the “Langevin Dynamics” page. As described above, Langevin dynamics mimics the frictional effects of solvent. CHARMMing is limited to 1,000 steps of dynamics, as described in the first article of this series. However, users have the option of downloading all generated inputs and running them locally, assuming that they have a CHARMM license. When submitting the Langevin dynamics calculation, the user is instructed to set the “FBETA” value to five. This controls the simulated collision frequency used in the dynamics, which simulates the jostling effects of solvent and also serves to couple the system to a heat bath (in CHARMMing, this is always set at 300 K). CHARMMing uses a 1 fs time step for all dynamics calculations; for a production run this may be increased for the reasons given above.

### Step 5: Perform Data Analysis

One of the most reliable metrics for gauging whether a protein is in a folded or unfolded state is the fraction of native contacts that are present (*Q*—see above for details). We have developed a native contacts calculator for G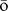 models in CHARMMing, and as the final step of the lesson, the user must use it to see how the fraction changes. Because the simulation is so short, native contacts are unlikely to change much, if at all; however, this is still a useful exercise since the same analysis can be applied to production simulations. The user is directed to visit the “KT-Go Native Contacts” page. Once he or she navigates to it, a table showing *Q* every 50 steps is displayed immediately; in the present version, no form needs to be filled in and submitted. At this point, all calculations for the lesson have been completed, and the user should have gained a very basic understanding of the methods and results.

## Discussion and Conclusion

CG models are widely used in the study of biophysics because of their ability to efficiently simulate biomolecules and qualitatively test hypotheses. The same attributes that make them suitable as research tools (their speed and their qualitative nature) also make them suitable as an education tool. While building and running CG models in simulation software previously required the expertise of a specialist in the field, this is no longer true, as the CHARMMing web portal to CHARMM now automates many of the tasks required to build a G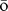 model, allowing this class of CG models to be used in the classroom.

CG G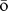 models and other reduced lattice models also have tremendous applications for research into the folding of small globular proteins, as they can correctly predict their thermodynamic properties [37], qualitative folding pathways [42], and ensembles of partially folded structures in the presence of various denaturing agents such as heat and chemicals [20]. The current work allows for the automation of the tedious process of parameter generation and model building for G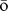 models. Analogous tools for AA simulation have proven very valuable to the modeling community [43].
